# Dietary Uridine Decreases Tumorigenesis in the *Apc^Min/+^* Model of Intestinal Cancer

**DOI:** 10.1093/cdn/nzy013

**Published:** 2018-03-13

**Authors:** Martha S Field, Xu Lan, Denise M Stover, Patrick J Stover

**Affiliations:** 1 Division of Nutritional Sciences, Cornell University, Ithaca, NY; 2 Graduate Field of Biochemistry, Molecular and Cell Biology, Cornell University, Ithaca, NY

**Keywords:** uridine, deoxyuridine, thymidine, colon cancer, methotrexate, 5-fluorouracil, folate-mediated one-carbon metabolism

## Abstract

**Background:**

Dietary deoxyuridine and uridine have been shown to have opposing effects on neural tube defect (NTD) incidence in the serine hydroxymethyltransferase 1 (*Shmt1^+/^^–^*) mouse model of folate-responsive NTDs, which are mediated by changes in de novo thymidylate biosynthesis. Alterations in folate-mediated one-carbon metabolism that protect against NTDs increased cancer risk in some studies.

**Objective:**

This study examined the effects of the dietary pyrimidine nucleosides uridine, thymidine, or deoxyuridine on intestinal tumorigenesis in the *Apc^Min/+^* mouse model [a mouse model lacking one copy of the adenomatosis polypsis coli (APC) gene] of spontaneous intestinal tumor formation. This study also evaluated the effects of uridine and deoxyuridine in culture medium on antifolate efficacy in Caco-2 and HeLa cell lines.

**Methods:**

*Apc^Min/+^* male mice (*n* = 10–14/group) were fed folate-deficient diets containing uridine, thymidine, or deoxyuridine from weaning until 17 wk of age. Total intestinal tumors were analyzed and biomarkers of folate status and metabolism were measured, including plasma folate concentrations, colon uracil content, and SHMT1 concentrations.

**Results:**

*Apc^Min/+^* mice fed dietary uridine showed a 50% reduction in total intestinal tumors, but neither dietary deoxyuridine nor thymidine affected tumorigenesis. Dietary nucleoside supplementation also increased plasma folate concentrations in *Apc^Min/+^* mice, as has been observed in the *Shmt1^+/^^−^* mouse model. Neither uridine nor deoxyuridine in culture media affected antifolate efficacy in either HeLa or Caco-2 cell lines.

**Conclusions:**

Dietary uridine, which is teratogenic in mice, decreases intestinal tumor formation in the *Apc^Min/+^* mouse model. Dietary uridine mimics the effect of the common methylene tetrahydrofolate reductase (*MTHFR*) C677T variant in protecting against colorectal cancer, while contributing to the risk of NTDs.

## Introduction

**FIGURE 1:**
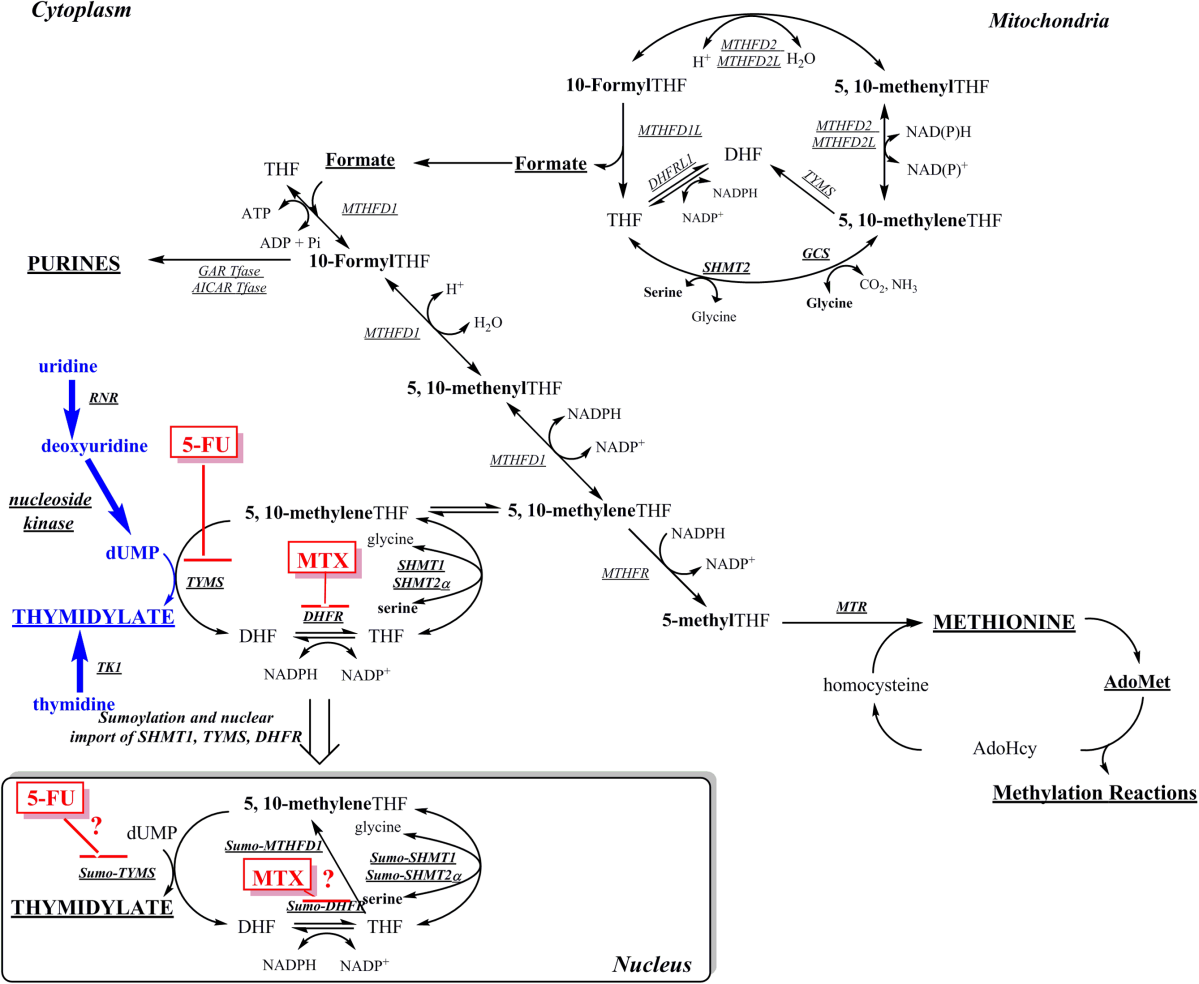
Folate-mediated one-carbon metabolism. Folate (THF) cofactors are required for the synthesis of purine nucleotides and dTMP and the remethylation of homocysteine to methionine for cellular methylation reactions. The dTMP biosynthesis pathway enzymes (MTHFD1, SHMT1, SHMT2α, TYMS, and DHFR) are SUMO-ylated and translocate to the nucleus during the S-phase. The remethylation of homocysteine to methionine is accomplished by methionine synthase, which requires vitamin B-12 as a cofactor. The activated one-carbon units are designated in bold. AdoHcy, *S*-adenosylhomocysteine; AdoMet, *S*-adenosylmethionine; AICAR, 5-aminoimidazole-4-carboxamide ribonucleotide; DHF, dihydrofolate; DHFR, dihydrofolate reductase; DHFRL1, dihydrofolate reductase-like 1; dTMP, thymidylate; dUMP, deoxyuridylate; GAR, glycinamide ribonucleotide; GCS, glycine cleavage system; MTHFD, methylenetetrahydrofolate dehydrogenase; MTHFR, methylenetetrahydrofolate reductase; MTR, methionine synthase; MTX, methotrexate; RNR, ribonucleotide reductase; SHMT, serine hydroxymethyltransferase; SUMO, small ubiquitin-like modifier; *Tfase*, transformylase; THF, tetrahydrofolate; TK, thymidine kinase; TYMS, thymidylate synthase; 5-FU, 5-fluorouracil; 10-FormylTHF, 10-formyltetrahydrofolate; 5,10-methenylTHF, 5,10-methenyltetrahydrofolate; 5,10-methyleneTHF, 5,10-methylenetetrahydrofolate.

Folate-mediated one-carbon metabolism (FOCM) is required for the de novo synthesis of 3 of the 4 deoxyribonucleotides required for DNA synthesis and for the remethylation of homocysteine to methionine (**Figure 1**) (1). Impaired FOCM is associated with pathologies, including a class of birth defects known as neural tube defects (NTDs, which result from the failure of neural tube closure during development), neurological disorders, and several types of cancer (1). Folic acid supplementation and fortification of the food supply prevent the majority of NTD cases in humans, but the mechanisms and associated pathways within this interconnected metabolic network that cause or prevent pathologies remain largely unknown (2). There have been suggestions in the literature that elevated folic acid is cancer promoting, but a 2013 meta-analysis including >50,000 individuals showed that folic acid intake at amounts that greatly exceed intake achieved in folic acid–fortified populations was not associated with an increased risk of several types of cancer (3). However, controversy remains in the field that, by stimulating cell growth and cell division, high amounts of folic acid intake may increase tumorigenesis in certain individuals (2, 4).

The de novo thymidylate (dTMP) synthesis pathway is particularly sensitive to perturbations in FOCM (1). The enzymes responsible for de novo dTMP synthesis comprise the thymidylate cycle (Figure 1). Thymidylate synthase converts deoxyuridylate and the folate cofactor 5,10-methylenetetrahydrofolate (5,10-methyleneTHF) to dTMP and dihydrofolate (DHF). DHF is converted to tetrahydrofolate (THF) by the enzyme DHF reductase; THF is then recycled back to 5,10-methyleneTHF by serine hydroxymethyltransferase (SHMT; SHMT1 or SHMT2α) using serine as a one-carbon source. Alternatively, 5,10-methyleneTHF can be generated by methylenetetrahydrofolate dehydrogenase 1 using formate as the one-carbon source (1). The enzymes that comprise the dTMP cycle have been shown to form a multienzyme complex, which colocalizes with the DNA replication machinery, such that dTMP is synthesized at sites of DNA synthesis (5). Moreover, SHMT1, SHMT2α, (the nuclear/cytosplasmic isoform of SHMT2) or both are required to anchor the dTMP synthesis machinery to nuclear lamin proteins and nuclear DNA (5, 6). There is also a salvage pathway for dTMP synthesis, which involves the conversion of thymidine to dTMP, catalyzed by thymidine kinase 1.

The *Shmt1^+/^^–^* mouse is the only mouse model of sporadic, folate-responsive NTDs associated with the disruption of a folate-requiring enzyme, and the NTDs are found in embryos lacking ≥1 copy of *Shmt1*. The primary phenotype of the *Shmt1^+/^^–^* mouse is elevated uracil in liver and colon genomic DNA (7, 8). In a study investigating whether the incidence of folate-deficiency–associated NTDs in this model could be modified by dietary dTMP precursors, dietary deoxyuridine was found to rescue NTD incidence, whereas dietary uridine caused NTDs independent of embryonic genotype and dietary folate (9). When the *Shmt1^+^^–^^-^* mouse was crossed to the *Apc^Min/+^* mouse model [a mouse model lacking one copy of the adenomatosis polypsis coli (APC) gene], which spontaneously forms intestinal tumors, mice heterozygous for both *Shmt1* and the *Min* allele showed increased total intestinal tumors (8).

The common methylene tetrahydrofolate reductase (*MTHFR*) C677T polymorphism results in a single amino acid change in MTHFR, creating a thermolabile enzyme. This polymorphism has 2 effects: *1*) it decreases synthesis of 5-methylTHF, which makes more folate cofactor available for dTMP synthesis (10, 11), and *2*) lowers intracellular folate concentrations (12). The polymorphism is associated with the risk of NTDs (2), but conversely is protective against colon cancer in folate-sufficient individuals (2). These studies indicate that the changes in FOCM that increase the risk of NTDs may also be protective against colon cancer. Given that deoxyuridine supplementation of the *Shmt1*^+/−^ mouse model prevents NTDs, in this study the relation between dietary deoxyuridine, uridine, or thymidine and tumorigenesis in the *Apc^Min/+^* model was investigated. The goal of this study was to determine if dietary pyrmidine nucleosides exhibited opposing effects on NTD incidence [previously published (9)] and colon cancer risk as observed with the *MTHFR* C677T polymorphism.

## Methods

### Diet studies

All animal experiments were approved by the Cornell Institutional Animal Care and Use Committee (Cornell University, Ithaca, New York) according to the guidelines of the Animal Welfare Act and all applicable federal and state laws. Mice were housed under specific-pathogen–free conditions in the Cornell University Weill Hall Barrier Facility, and food and water were provided ad libitum. C57Bl/6.*Apc^Min/+^* male mice were generated by crossing C57Bl/6.*Apc^Min/+^* male mice (Jackson Laboratories) to C57Bl/6 female mice. Genotyping of the *Apc^Min^* allele was carried out as previously described (13). Male C57Bl/6.*Apc^Min/+^* mice (9–11/diet group) were randomly assigned to 1 of 7 modified AIN-93G diets (Dyets, Inc.) at weaning (3 wk of age) and maintained on the diets for 14 wk. All diets have been described previously (9) and were based on a modified AIN-93G diet lacking folate with varying amounts of nucleosides: 0 nucleosides [“control”; folate deficient (FD)], 0.6% (wt:wt) uridine (FD+U), 0.2% thymidine (FD+T), 0.1% deoxyuridine (FD+dU), 0.1% uridine, 0.01% uridine, and 0.001% uridine. These diets did not contain antibiotics. None of the study mice were lost before they were killed at 17 wk of age.

### Tissue collection and tumor evaluation

Mice were killed by cervical dislocation. Blood was collected by cardiac puncture into EDTA-coated tubes. Plasma was separated from RBCs by centrifugation. Livers and kidneys were removed, washed in cold PBS, snap-frozen in liquid nitrogen, and then stored at –80C. The small intestine was removed, divided into 3 sections (duodenum, jejunum, and ileum), flushed with cold PBS, and opened longitudinally. Intestinal sections were placed lumen side up, and tumor number and size were then measured using a dissecting microscope as previously described (13). The colon was treated similarly, with the exception that it was divided longitudinally into 3 equally sized sections after analysis and snap-frozen. Tumor burden was quantified as previously described (8).

### Plasma and tissue folate concentration

Folate concentrations of plasma and tissue were quantified by using the *Lactobacillus casei* microbiological assay as described previously (14), with the following modifications for liver and colon tissue: 500 mg liver or 1 colon section (the proximal one-third and free of tumors) was sonicated in 100 µL of a buffered solution containing 2% (wt:vol) sodium ascorbate, 0.2 M 2-mercaptoethanol, 0.05 M HEPES, and 0.05 M N-Cyclohexyl-2-aminoethanesulfonic acid (pH 7.85). Samples were then centrifuged in a 4°C microcentrifuge (Eppendorf) for 5 min at maximum speed. Twenty-five microliters of supernatant was removed for determination of total protein concentration (15), and the remaining supernatant was boiled for 10 min, cooled for 10 min on ice, and then centrifuged in a 4°C microcentrifuge for 5 min at 14000 × *g*. The supernatant was then treated with rat plasma conjugase, and total folates (normalized to total protein in each tissue sample) were determined as described previously (14).

### Effects of nucleotide supplementation on IC_50_ values for 5-fluorouracil and methotrexate in HeLa cells

HeLa and Caco-2 cells (passages 24–26) were regularly passaged in minimal essential medium α-modification (Hyclone) supplemented with 10% FBS (Hyclone) and 1% penicillin/streptomycin (Corning). For experiments to determine concentration of an inhibitor where the response is reduced by half (IC_50_), of antifolates, cells were cultured in defined minimal essential medium α-modification that lacks ribonucleotides, deoxyribonucleotides, folate, pyridoxine, methionine, serine, and glycine and supplemented with 10% (vol:vol) dialyzed, charcoal-treated FBS (Hyclone), 200 µM methionine, 1 mg pyridoxine/L, and 2 µM folic acid. HeLa cells were seeded into 96-well plates at densities of 2000 cells/well, and Caco-2 cells were plated at 5000 cells/well. Immediately after seeding, cells were treated with 50 µM deoxyuridine or 50 µM uridine, and 6 concentrations of 5-fluorouracil (5-FU; 0–1000 µM; Sigma) or methotrexate (MTX; 0–0.5 µM; Sigma) with *n* = 10 wells per treatment condition and cultured for 96–120 h. Cell viability was measured by using the 3-(4,5-dimethylthiazol-2-yl)-2,5-diphenyltetrazolium bromide (MTT) assay and IC_50_ values were calculated with the use of Prism software as previously described (16).

### Immunoblotting

The middle one-third section of the colons (all of which were free of tumors) were lysed by sonication in a solution containing 10 mM Tris, pH 7.4; 150 mM NaCl; 5 mM EDTA; 5 mM DTT; 1% Triton X-100; and Mammalian Protease Inhibitor Cocktail (Sigma). Lysates were loaded onto 10% SDS-PAGE gels with 40 µg total protein/lane. Protein concentrations were determined with the use of the Lowry-Bensadoun method (15). Proteins were then transferred to an Immobilon-P polyvinylidene difluoride membrane (Millipore). The membrane was blocked overnight at 4°C in PBS with 10% nonfat dry milk and 1% NP40. The membrane was incubated at 4°C overnight in a purified antibody (1:10,000 dilution) of sheep α-SHMT1 [described previously by Liu et al. (17)]. After 4 washes of 10 min each, the membrane was incubated for 2 h in 1:10,000 HRP-conjugated goat α-sheep antibody (Pierce). After 4 washes of 10 min each, membranes were developed in SuperSignal West Pico Chemiluminescent Substrate (Pierce) and films exposed. As a loading control, membranes were also probed with an antibody to GAPDH (Novus Biologicals) used at 1:200,000 dilution; HRP-conjugated goat anti-mouse secondary antibody (Pierce) was used at 1:10,000 dilution. Images were digitized and quantification of protein expression was performed with the use of Image J software (NIH).

### Detection of uracil in nuclear DNA

Uracil content in colonic nuclear DNA was determined by using GC-MS as previously described (7).

### Statistical analysis

Differences between groups were determined by using a Student's *t* test, with a Bonferroni correction for a prespecified number of multiple comparisons. Comparisons were considered significant at *P *< 0.05.

## Results

### Dietary uridine reduces total intestinal tumor number in *Apc^Min/+^* mice

**FIGURE 2:**
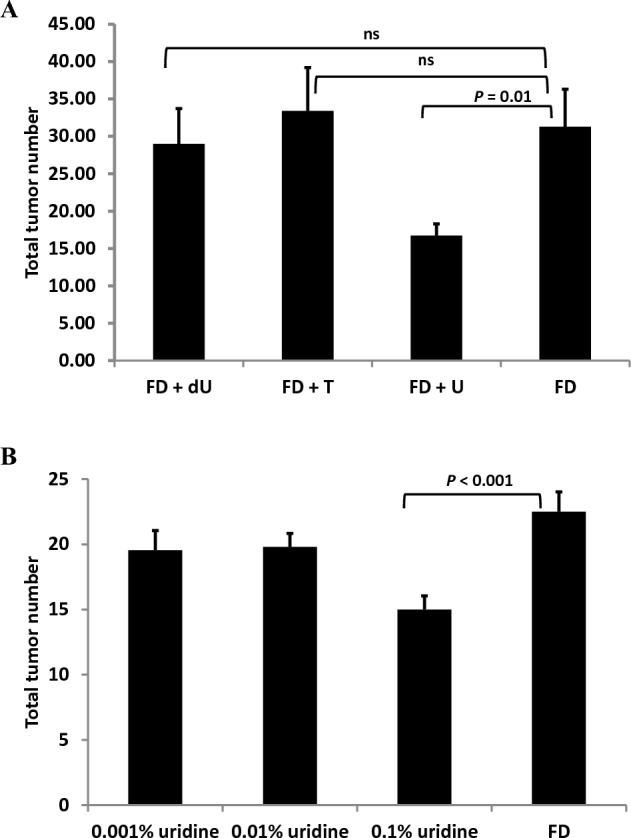
Dietary uridine decreases mean intestinal tumor number in *Apc^Min/+^* mice fed an AIN-93G diet. Values are total tumor numbers in the small intestine and colon ± SEMs, *n* = 9–11/group. Data were analyzed by using a Student's *t* test with a Bonferroni correction for multiple comparisons. (*A*) Mice were weaned onto a nucleoside-containing folate-deficient AIN-93G (0.6% uridine, 11 mice; 0.2% thymidine, 10 mice; 0.1% deoxyuridine, 9 mice) or FD control (10 mice) diet at 3 wk of age and consumed the diets until being killed at 17 wk of age. (B) Mice were weaned onto a uridine-supplemented folate-deficient AIN-93G (0.1% uridine, 12 mice; 0.01% uridine, 10 mice; 0.001% uridine, 11 mice) or FD control (9 mice) diet at 3 wk of age and consumed the diets until being killed at 17 wk of age. *Apc^Min/+^*, mouse model lacking one copy of the APC gene; dU, deoxyuridine; FD, folate-deficient; T, thymidine; U, uridine.

**FIGURE 3:**
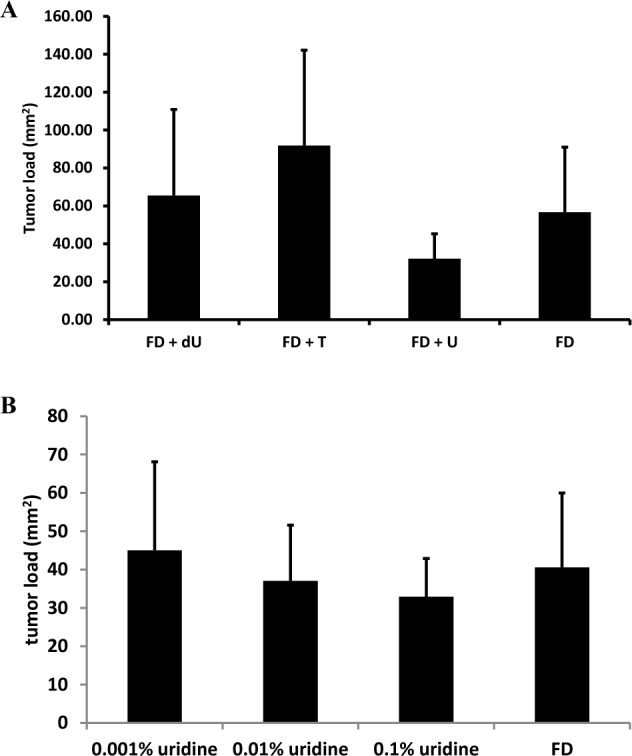
Dietary uridine affects mean intestinal tumor burden in *Apc^Min/+^* mice fed an AIN-93G diet. Values are total tumor burden in the small intestine ± SDs, *n* = 9–11/group. Data were analyzed by using a Student's *t* test with a Bonferroni correction for multiple comparisons. (A) Mice were weaned onto a nucleoside-containing folate-deficient AIN-93G (0.6% uridine, 11 mice; 0.2% thymidine, 10 mice; 0.1% deoxyuridine, 9 mice) or FD control (10 mice) diet at 3 wk of age and consumed the diets until being killed at 17 wk of age. (B) Mice were weaned onto a uridine-supplemented folate-deficient AIN-93G (0.1% uridine, 12 mice; 0.01% uridine, 10 mice; 0.001% uridine, 11 mice) or FD control diet (9 mice) at 3 wk of age and consumed the diets until being killed at 17 wk of age. *Apc^Min/+^*, mouse model lacking one copy of the APC gene; dU, deoxyuridine; FD, folate-deficient; T, thymidine; U, uridine.

Male *Apc^Min/+^* mice were fed folate-deficient AIN-93G–based diets supplemented with nucleosides deoxyuridine, thymidine, or uridine from weaning to 17 wk of age as described in Methods. More than 95% of tumors in mice fed all diets were in the small intestine, with each mouse exhibiting 1 tumor (or no tumors) in the colon. Supplementation with 0.6% (wt:wt) dietary uridine (FD+U) reduced total intestinal tumor number (small intestine and colon) by nearly 50% relative to the folate-deficient AIN-93G (FD) control diet (**Figure 2A**). Neither dietary deoxyuridine (FD+dU diet) nor thymidine (FD+T diet) affected total intestinal tumor number (Figure 2A). *Apc^Min/+^* mice fed the FD diet supplemented with 0.1% (wt:wt) uridine also showed a 30% decrease in total intestinal tumors relative to *Apc^Min/+^* mice fed the FD diet, indicating a dose-response relation between dietary uridine exposure and intestinal tumor number (Figure 2B). Tumor load is a measure of total tumor area per animal and serves as an additional measure of severity of tumor progression. Trends similar to those observed for the effect of dietary nucleosides on tumor number were also observed for tumor load, but most comparisons did not withstand correction for multiple comparisons (**Figure 3**).

### Dietary nucleoside supplementation increases plasma folate concentrations

**FIGURE 4:**
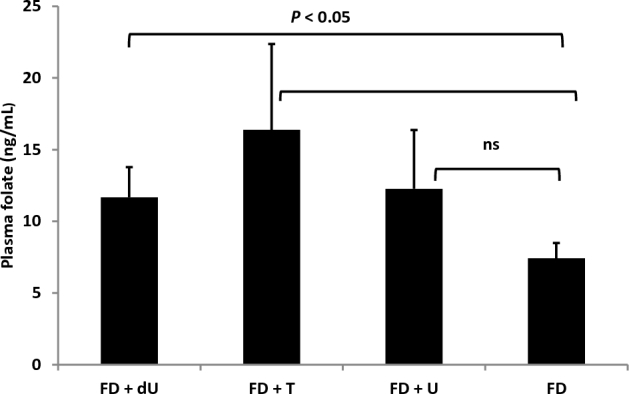
Dietary nucleosides increase mean plasma total folate concentration. Values are means ± SEMs; *n* = 4–6 mice/dietary intervention group (FD+U, 5 mice; FD+T, 5 mice; FD+dU, 4 mice) or *n* = 5 mice for the FD control diet, with significance determined by using a Student's *t* test with a Bonferroni correction for multiple comparisons. dU, deoxyuridine; FD, folate-deficient; T, thymidine; U, uridine.

In a previous study of the effects of dietary nucleosides on NTD incidence, dietary deoxyuridine and thymidine increased plasma total folate concentrations in the *Shmt1^+/^^−^* mouse model on a *129SvEv* background (9). In this study in the *Apc^Min/+^* model on a C57Bl/6 background, mice fed deoxyuridine and thymidine also exhibited a trend toward increased plasma total folate concentrations relative to plasma folate concentrations in mice fed the FD diet (**Figure 4**). Liver and colon total folate concentrations were not different among nucleoside-supplemented diets (data not shown).

### SHMT1 expression in the colon is not affected by dietary nucleosides

**FIGURE 5:**
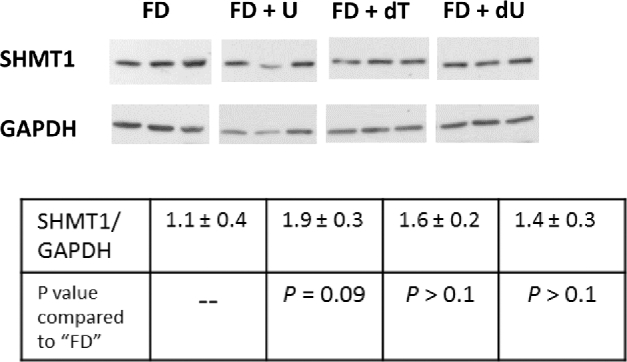
SHMT1 expression in the colon does not differ among nucleoside-containing diets. Protein expression concentrations vary longitudinally along the colon and in tumor tissue; therefore, sections from the middle one-third of the colon and free of tumors (*n* = 3/group free of tumors) were removed and proteins were extracted. Proteins were separated on a 10% SDS-PAGE gel, then transferred to a polyvinylidene difluoride membrane for Western blot analysis. Densitometry was performed by using ImageJ software, and the intensity of the SHMT1 protein band was normalized to the intensity of GAPDH for each sample. Values are means ± SDs, *n* = 3 samples/group. Significance was determined by using a Student's *t* test with a Bonferroni correction for multiple comparisons. There were no differences among SHMT1 expression in colons of mice fed the FD AIN-93G-based control diet and the mice fed any of the nucleoside-containing diets. dT, thymidine; dU, deoxyuridine; FD, folate-deficient; SHMT, serine hydroxymethyltransferase; U, uridine.

**FIGURE 6:**
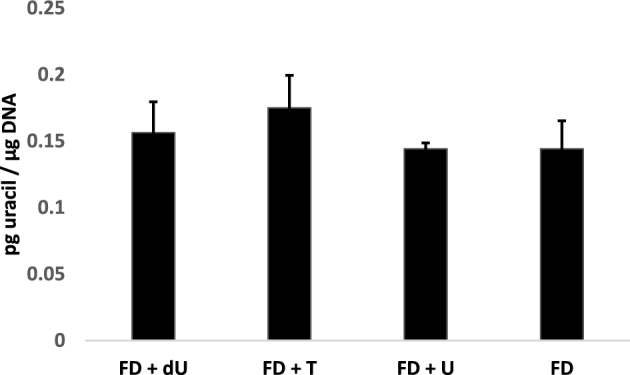
Dietary nucleoside supplementation does not affect uracil concentrations in colon DNA. Genomic DNA was isolated from the proximal one-third of each colon section (free of tumors), and uracil in DNA was measured by using GC-MS as described in Methods. Values are means ± SEMs, *n* = 3–5 mice/diet group (FD+U, 4 mice; FD+T, 3 mice; FD+dU, 5 mice) or *n* = 4 mice for the FD control diet.

SHMT1 protein concentrations in the colon did not differ as a function of dietary nucleosides (**Figure 5**). Similarly, uracil concentrations in colon DNA did not change a result of exposure to nucleosides (**Figure 6**), an observation that is consistent with previous studies that quantified uracil in liver DNA from pregnant dams fed the same diets (9).

### Uridine and deoxyuridine minimally modify MTX and 5-FU toxicity

The addition of uridine or deoxyuridine in culture media did not have biologically meaningful effects on the cytotoxicity of antifolates in cell culture models (Caco-2 and HeLa). The IC_50_ for 5-FU and MTX in Caco-2 cells was not affected by the addition of nucleosides to culture media (**Table 1**). In HeLa cells, nucleosides in culture media minimally affected IC_50_ for these compounds. Uridine exposure increased the IC_50_ of HeLa cells for 5-FU by 30% and for MTX by 16% (**Table 2**). Conversely, deoxyuridine exposure decreased the IC_50_ for 5-FU by 23% and by 15% for MTX (Table 2). Similarly, neither deoxyuridine nor uridine in culture medium affected Caco-2 growth rate or caspase activation (1 measure of apoptosis; data not shown).

**TABLE 1:** IC_50_ values in Caco-2 cells cultured for 96–120 h with 50 µM deoxyuridine or 50 µM uridine^1^

	IC_50_ relative to control (no nucleosides)		
Treatment	50 µM uridine	50 µM deoxyuridine	*P* (comparison between nucleotide treatments)
5-Fluorouracil	0.88 ± 0.18	0.91 ± 0.31	>0.1
Methotrexate	0.93 ± 0.03	0.86 ± 0.03	0.06

1 Values are means ± SDs of the normalized ratio of 2 independent experiments. IC_50_ curves were generated by using 0.1–1000 µM 5-fluorouracil and 0.001–0.5 µM methotrexate; viability was measured by using the MTT assay. IC_50_ values for each treatment were normalized to IC_50_ of control (no nucleoside supplementation). IC_50_, concentration of an inhibitor where the response is reduced by half; MTT, 3-(4,5-dimethylthiazol-2-yl)-2,5-diphenyltetrazolium bromide.

**TABLE 2:** IC_50_ values in HeLa cells cultured for 96–120 h with 50 µM deoxyuridine or 50 µM uridine^1^

	IC_50_ relative to control (no nucleosides)		
Treatment	50 µM uridine	50 µM deoxyuridine	*P* (comparison between nucleotide treatments)
5-Fluorouracil	1.30 ± 0.18	0.77 ± 0.20	*P* < 0.01
Methotrexate	1.16 ± 0.12	0.85 ± 0.26	*P* = 0.03

1 Values are means ± SDs of the normalized ratio of 4 independent experiments. IC_50_ curves were generated by using 0.1–1000 µM 5-fluorouracil and 0.001–0.5 µM methotrexate; viability was measured by using the MTT assay. IC_50_ values for each treatment were normalized to IC_50_ of control (no nucleoside supplementation). IC_50_, concentration of an inhibitor where the response is reduced by half; MTT, 3-(4,5-dimethylthiazol-2-yl)-2,5-diphenyltetrazolium bromide.

## Discussion

The *Shmt1*^+/−^ mouse model is sensitized to folate-responsive NTDs and causes increased intestinal tumor formation when crossed to the *Apc^Min/+^* mouse model (8). Interestingly, NTD incidence in the *Shmt1^+/-^* mouse model is rescued by either dietary folate or dietary deoxyuridine, whereas dietary uridine increased NTDs in mice, independent of dietary folic acid and *Shmt1* genotype (9). It has been proposed that deoxyuridine rescues NTDs and accelerates dTMP synthesis by providing increased concentrations of the deoxyuridylate substrate for thymidylate synthase–catalyzed formation of dTMP (Figure 1). The mechanism whereby dietary uridine induced NTDs is not understood but may be independent of folate metabolism, because neither folate status nor *Shmt1* genotype influenced its teratogenicity.

The purpose of this study was to determine if nucleoside supplementation influenced cancer outcomes in the *Apc^Min/+^* mouse model and to see if the pathways and underlying mechanisms for NTDs and colon tumorigenesis were shared. Surprisingly, neither deoxyuridine nor thymidine supplementation affected tumorigenesis in the *Apc^Min/+^* mouse model, whereas 0.6% uridine supplementation decreased total intestinal tumors by 50% (Figure 2A). The change in total tumor number as a result of uridine supplementation in the *Apc^min/+^* model is on the same order of magnitude as the change induced by deletion of the *c-Myc* oncogene, which is known to function in APC-mediated carcinogenesis (18). There was also a dose-response relation between uridine supplementation and total intestinal tumors, in that supplementation with 0.1% uridine resulted in a 30% decrease in tumors (Figure 2B). Although the amounts of nucleosides added to these AIN-93G–based diets are relatively high, previous studies have shown that nucleoside bioavailability in rodents is relatively low (19–21). Furthermore, in a previous study that used the same diet compositions, no change in plasma nucleoside concentrations was observed in response to these diets (9). Interestingly, in this study and a previous study (9), dietary nucleosides did not significantly increase plasma nucleoside concentrations. There was, however, an interaction between plasma folate concentrations and dietary nucleosides. Dietary thymidine and deoxyuridine increased plasma folate by almost 60% in female mice on a *129SvEv* background (9). In this study in male *Apc^Min/+^* mice on a C57Bl/6 background, plasma folate concentrations increased by 50% in response to deoxyuridine supplementation, and there was a trend toward 2-fold increased plasma folate concentrations with thymidine that did not withstand correction for multiple comparisons (Figure 4). This suggests that dietary nucleosides affect folate homeostasis, but the increase in plasma folate occurred without changes in liver folate concentrations, so it is not clear what might be driving this change in whole-body folate distribution. The mechanisms and physiologic significance of the effect of dietary deoxyuridine and other pyrmidine nucleosides on plasma folate concentrations remain to be determined.

*Shmt1^+/^^−^Apc^Min/+^* mice fed FD diets have increased total intestinal tumors and increased uracil in colon DNA relative to *Shmt1^+/+^Apc^Min/+^* mice, suggesting a relation between *Shmt1* heterozygosity, folate status, uracil in DNA, and intestinal tumorigenesis (13). Uracil in DNA results from impaired de novo dTMP synthesis, which is caused either by folate deficiency or lack of SHMT1. SHMT1 is essential for de novo dTMP synthesis because it anchors the de novo dTMP synthesis complex to the DNA and facilitates interaction of the complex with the DNA repair machinery (5). Nuclear localization of SHMT1 has been shown to be critical for limiting uracil in nuclear DNA (7, 22). This raises the possibility that dietary uridine may affect intestinal tumorigenesis by affecting intestinal SHMT1 expression or uracil in DNA. However, Western blot showed that SHMT1 expression in the colon was not affected by dietary nucleosides (Figure 5). Similarly, dietary nucleosides did not affect uracil concentrations in colon DNA (Figure 6). These data suggest that dietary uridine may reduce intestinal tumor formation through folate-independent mechanisms, mirroring previous findings that uridine supplementation causes NTDs independent of folate metabolism (9). Observational studies have shown that nuclear localization of the dTMP synthesis pathway modifies response to 5-FU in human colorectal cancer (23, 24), and MTX has been shown to block DHF-reductase nuclear translocation, suggesting an interaction between nuclear localization of the dTMP synthesis pathway and antifolate drug response. The observation that neither deoxyuridine nor uridine in culture medium has a meaningful pharmacologic effect on the toxicity of 2 classical antifolate compounds (5-FU and MTX) in 2 cancer cell lines further supports the hypothesis that uridine affects tumorigenesis through folate-independent mechanisms.

The effect of uridine availability on genome stability is not clear in the literature. Mice lacking uridine phosphorylase (*UPase^−^^/^^−^*), which reversibly interconverts uridine and uracil, accumulate 6-fold more uridine in plasma than wild-type mice (6). There is also accumulation of uridine and alterations in nucleotide pools in some tissues including liver, gut, and kidney, but no effects on growth or histology of major organs at 5 mo of age (6). A recent study indicated increased DNA damage in the lung, liver, colon, and stomach of *UPase^−^^/^^−^* mice (25). In cancer cell lines cultured with 10 µM and 30 µM uridine, there was also an increase in DNA strand breaks, which the authors propose is the result of increased uracil in DNA (25). Uracil concentrations in DNA were not directly measured in this study, because the fragment length analysis using repair enzymes assay measures DNA strand breaks resulting either from repair of uracil incorporated into DNA or DNA strand breaks resulting from stalled replication forks, which can be caused by nucleotide pool imbalances (26). With the use of a more specific GC-MS–based assay for uracil in DNA, previous studies in HeLa cells indicated that uracil concentrations in DNA did not increase after being cultured for 4 doublings in cell culture medium supplemented with 50 µM uridine (9). Similarly, in this study, uracil in colon DNA did not increase as a result of uridine supplementation in *Apc^Min/+^* mice (Figure 6). Overall, studies seem to indicate that uridine exposure increases genome damage but does not influence uracil accumulation in DNA.

This study was powered to detect differences in total intestinal tumor number as a result of exposure to dietary nucleosides. Protein expression levels vary longitudinally in colon tissue, necessitating that biomarkers are measured within given longitudinal sections (i.e., protein expression measurements were performed in the middle one-third of the colon). In addition, some colon tissue sections also contained tumors, further limiting the number of colon sections that could be used for molecular studies such as uracil in DNA, total folate measurement, and SHMT1 protein concentrations in colon tissue. One limitation of the study is that statistical power was limited with respect to measurement of biomarkers indicative of mechanism. Further research is required to understand the relations between nucleoside exposure, uracil in DNA, DNA damage, and replication fork stalling and cancer outcomes.

Dietary uridine mimics the effects of the *MTHFR* 677T variant, which decreases colorectal tumorigenesis but increases the risk of NTDs (9). The contrasting effects of dietary uridine and deoxyuridine in 2 different mouse models of human pathology, colon cancer, and folate-responsive NTDs indicate a potential shared etiology and pathophysiologic mechanism. Uridine triacetate was recently approved by the FDA as a treatment to rescue 5-FU and capecitabine toxicity, presumably by rescuing toxicity due to the incorporation of a 5-FU metabolite, 5-FUTP, into RNA (27). To our knowledge, to date there are no studies indicating whether uridine triacetate also affects tumorigenesis in humans, or whether it is teratotogenic, as observed in mice (9).

## Notes

Supported by Public Health Service grant R37DK58144 to PJS.

Author disclosures: MSF, XL, DMS, and PJS, no conflicts of interest.

Abbreviations used:

APC: adenomatosis polyposis coli
APCMin/+: mouse model lacking one copy of the APC gene
DHF: dihydrofolate
dTMP: thymidylate
FD: folate deficient
FOCM: folate-mediated one-carbon metabolism
IC_50_: concentration of an inhibitor where the response is reduced by half
MTT: 3-(4,5-dimethylthiazol-2-yl)-2,5-diphenyltetrazolium bromide
MTX: methotrexate
NTD: neural tube defect
SHMT: serine hydroxymethyltransferase
THF: tetrahydrofolate
5-FU: 5-fluorouracil
5,10-methyleneTHF: 5,10-methylenetetrahydrofolate
